# Targeting hepatic pyruvate dehydrogenase kinases restores insulin signaling and mitigates ChREBP-mediated lipogenesis in diet-induced obese mice

**DOI:** 10.1016/j.molmet.2018.03.014

**Published:** 2018-03-31

**Authors:** Cheng-Yang Wu, Shih-Chia Tso, Jacinta L. Chuang, Wen-Jun Gui, Mingliang Lou, Gaurav Sharma, Chalermchai Khemtong, Xiangbing Qi, R. Max Wynn, David T. Chuang

**Affiliations:** 1 Department of Biochemistry, University of Texas Southwestern Medical Center, Dallas, TX, USA; 2 Department of Internal Medicine, University of Texas Southwestern Medical Center, Dallas, TX, USA; 3 Advanced Imaging Research Center, University of Texas Southwestern Medical Center, Dallas, TX, USA; 4 Chemistry Center, National Institute of Biological Science, Beijing, China; 5 Graduate School of Peking Union Medical College and Chinese Academy of Medical Sciences, Beijing, China

**Keywords:** Pyruvate dehydrogenase kinase inhibitor, Liver, Glucose homeostasis, Insulin sensitivity, Hepatic steatosis, ChREBP

## Abstract

**Objective:**

Mitochondrial pyruvate dehydrogenase kinases 1–4 (PDKs1–4) negatively regulate activity of the pyruvate dehydrogenase complex (PDC) by reversible phosphorylation. PDKs play a pivotal role in maintaining energy homeostasis and contribute to metabolic flexibility by attenuating PDC activity in various mammalian tissues. Cumulative evidence has shown that the up-regulation of PDK4 expression is tightly associated with obesity and diabetes. In this investigation, we test the central hypothesis that PDKs1-4 are a pharmacological target for lowering glucose levels and restoring insulin sensitivity in obesity and type 2 diabetes (T2D).

**Methods:**

Diet-induced obese (DIO) mice were treated with a liver-specific pan-PDK inhibitor 2-[(2,4-dihydroxyphenyl) sulfonyl]isoindoline-4,6-diol (PS10) for four weeks, and results compared with PDK2/PDK4 double knockout (DKO) mice on the same high fat diet (HFD).

**Results:**

Both PS10-treated DIO mice and HFD-fed DKO mice showed significantly improved glucose, insulin and pyruvate tolerance, compared to DIO controls, with lower plasma insulin levels and increased insulin signaling in liver. In response to lower glucose levels, phosphorylated AMPK in PS10-treated DIO and HFD-fed DKO mice is upregulated, accompanied by decreased nuclear carbohydrate-responsive element binding protein (ChREBP). The reduced ChREBP signaling correlates with down-regulation of hepatic lipogenic enzymes (ACC1, FAS, and SCD1), leading to markedly diminished hepatic steatosis in both study groups, with lower circulating cholesterol and triacylglyceride levels as well as reduced fat mass. PS10-treated DIO as well as DKO mice showed predominant fatty acid over glucose oxidation. However, unlike systemic DKO mice, increased hepatic PDC activity alone in PS10-treated DIO mice does not raise the plasma total ketone body level.

**Conclusion:**

Our findings establish that specific targeting of hepatic PDKs with the PDK inhibitor PS10 is an effective therapeutic approach to maintaining glucose and lipid homeostasis in obesity and T2D, without the harmful ketoacidosis associated with systemic inhibition of PDKs.

## Highlights

• Diet-induced obese (DIO) mice were treated with a novel liver-specific pyruvate dehydrogenase kinase (PDK) inhibitor PS10.
• PS10-treated DIO mice and PDK2/PDK4-DKO mice fed indentical HFD showed improved glucose, insulin and pyruvate tolerance.
• PS10-treated DIO and untreated DKO mice manifest reduced hepatic steatosis and attenuation of ChREBP-mediated lipogenesis.
• Unlike DKO mice, targeting hepatic PDK alone in DIO mice with PS10 does not increase total plasma ketone-body levels.

## 1 Introduction

The mitochondrial pyruvate dehydrogenase complex (PDC), which converts pyruvate to acetyl-CoA via oxidative decarboxylation, is acutely regulated by reversible phosphorylation (inactivation) and dephosphorylation (activation) catalyzed by the bound pyruvate dehydrogenase kinases (PDKs 1–4) and pyruvate dehydrogenase phosphatases (PDPs 1–2) [1], respectively. PDKs play a pivotal role in maintaining energy homeostasis and contribute to metabolic flexibility by attenuating PDC activity in various mammalian tissues [2]. Cumulative evidence has shown that the up-regulation of PDK4 expression is tightly associated with obesity [3], [4] and diabetes [5]. PDK4 mediates lipogenesis and also contributes to the pathogenesis of nonalcoholic steatohepatitis [6]. PDK4 is upregulated by glucocorticoids through FOXO transcription factors, which are inactivated by insulin upon phosphorylation by Akt [7], [8]. Metformin suppresses growth hormone-mediated PDK4 expression via an AMP-activated protein kinase (AMPK)-SHP pathway [9] and improves fatty liver disease in *ob/ob* mice [10]. PDK4 knockout (KO) [11], [12] and PDK2/PDK4 double knockout (DKO) mice [13] exhibit increased glucose oxidation in skeletal muscle with significantly better glucose tolerance and insulin sensitivity. These PDK-deficient mice also showed body weight loss and reduced lipogenesis manifested by markedly diminished hepatic steatosis, compared to wild-type mice on the same high-fat diet [11]. The mechanism for the correlation between increased glucose oxidation and reduced hepatic lipogenesis remains poorly understood.

Hepatic lipogenesis can be controlled by insulin signaling primarily through the sterol regulatory element-binding protein 1c (SREBP-1c) [14]. The transcription factor promotes expression of several enzymes that are important for *de novo* lipogenesis, including acetyl-CoA carboxylase 1 (ACC-1), fatty acid synthase (FAS), stearoyl-CoA desaturase 1 (SCD1), and long-chain fatty acid elongase (LCE) [15]. In addition, glucose can directly stimulate lipogenesis through the activation of another basic helix-loop-helix zipper transcription factor known as the carbohydrate-response element-binding protein (ChREBP) in liver. High concentrations of glucose in the cytoplasm cause ChREBP to be translocated into the nucleus. A primary enzyme up-regulated by nuclear ChREBP is the cytosolic liver-type pyruvate kinase (L-PK), which catalyzes the conversion of phosphoenolpyruvate to pyruvate [16], [17]. ChREBP tightly couples metabolism of excess carbohydrates with hepatic fat synthesis for energy storage by increasing the expression of lipogenic enzymes in an insulin-independent manner. The mRNA of a nuclear ChREBP isoform (ChREBP-β) was isolated from the white adipose tissue (WAT) [18], [19].

To test the central hypothesis that PDKs1-4 are a pharmacological target for obesity and type 2 diabetes, our laboratory recently developed novel pan-PDK inhibitors 2-[(2,4-dihydroxyphenyl)sulfonyl]isoindoline-4,6-diol (PS10) [20] and (S)-3-amino-4-(4-((2-((2,4-dihydroxyphenyl)sulfonyl)isoindolin-5-yl)-amino)piperidin-1-yl)-4-oxobutanamide (compound 17 or PS46) [21]. Unlike the systemic allosteric PDK inhibitor dichloroacetate (DCA), PS10 and PS46 bind to the active site and shows preferential inhibition of PDK activity in liver [20], [21]. In the present study, we investigated mechanisms for improved glucose tolerance and reduced hepatic steatosis in PS10-treated (DKO) mice and PDK2/PDK4 double knockout mice fed a high-fat diet (HFD).

## 2 Methods

### 2.1 Animals

C57BL/6J male mice at six-to eight-weeks old were obtained from the local campus-breeding colony at UT Southwestern Medical Center (Dallas, TX). Mice were fed with a 60% high-fat diet, which contained 32% saturated and 68% unsaturated fat (catalog number: D12492, Research Diet Inc. New Brunswick, NJ), for 18 weeks to produce DIO animals. Mice were housed in an air-conditioned animal facility with 12-hour light-to-dark cycle. The protocol of accommodation and treatment was approved by IUCAC committee at UT Southwestern Medical Center.

### 2.2 Sample analysis of diet-induced obese mice treated with PDK inhibitor

Blood was harvested by cardiac puncture and stored on ice. Acidified citrate dextrose was used as an anticoagulant. Immediately after blood collection, heart, liver, kidneys, and both hind-leg quadriceps muscles were removed and snap frozen in liquid nitrogen. Average ischemia time before organ harvest was about 2–3 min. PDC activities in mouse tissues were assayed as described previously [20]. Blood was centrifuged in an Eppendorf 5415R refrigerated microcentrifuge at 9,300 × g for 10 min to isolate plasma; the latter was subsequently stored at −80 °C.

### 2.3 Glucose tolerance test

Mice were fasted for 6 h following PS10 treatment. Eight hours after PS10 administration, 1.5 g/kg of glucose was delivered intraperitoneally to mice. Tail vein serum samples were collected immediately before and 15, 30, 60, and 120 min after the glucose challenge. Plasma glucose concentrations (mg/dL) were determined using glucose strips in a BAYER glucose meter for all tolerance tests.

### 2.4 Insulin tolerance test

Mice were fasted for 3.5 h after PS10 treatment. Eight hours after PS10 administration, 0.75 U/kg of insulin was delivered intraperitoneally to mice. Tail vein serum samples were collected immediately before and 15, 30, 60, and 120 min after the insulin challenge.

### 2.5 Pyruvate tolerance test

Mice were fasted for 6 h after PS10 treatment. Eight hours after PS10 administration, 2.0 g/kg of pyruvate sodium salt solution in 1X PBS was delivered intraperitoneally to mice. Tail vein serum samples were collected immediately before and 15, 30, and 60 min after the pyruvate challenge.

### 2.6 Metabolic cage study

*In vivo* metabolic profiling was assessed by using the PhenoMaster Home Cage System (TSE Systems International Groups). Mice were subjected to five-days acclimation before the measurements were initiated. Monitoring occurred over 5 days under a normal 12:12 h light:dark cycle. Measurements were taken at an interval of 46 min for energy parameters and activity parameters. Body composition was measured before the acclimation and after the 5-day measurement with the Echo MRI-100 spectrometer (Echo MRI LLC, Houston, USA).

### 2.7 Blood biochemistry

Plasma lactate, cholesterol, triglyceride, AST, and ALT were measured by Vitros 250 blood chemistry analyzer (Johnson & Johnson Inc.); the total ketone body and non-ester free fatty acid were measured by corresponding enzymatic assays in the Metabolic Phenotyping Core in UT Southwestern Medical Center. Plasma insulin levels were measured by RayBio® Mouse Insulin ELISA kit (ELM-Insulin, RayBiotech).

### 2.8 Liver histochemistry

Histological examination of the liver was performed in the institutional immunohistochemistry laboratory. Liver tissue was dissected, grossly trimmed then fixed by immersion for 48 h in 4% formalin/PBS (4% formic acid, 137 mM NaCl, 2.7 mM KCl and 10 mM phosphate buffer, pH 7.5) at 4 °C. Liver samples were then transferred to 10% (w/v) sucrose in PBS and incubated at 4 °C for 24 h. Tissues were incubated in 18% sucrose in PBS at 4 °C for 24 h. Finally, samples were transferred to a fresh 18% sucrose solution and embedded in optimal cutting temperature compounds (OCT), cryo-sectioned, and stained with Oil Red O.

### 2.9 ChREBP DNA-binding activity assay

Gel-shift assay was performed to assess the LPK Carbohydrate-responsive element (ChRE)–binding activity to ChREBP in the nuclear extract. Wild-type LPK ChRE double-stranded oligonucleotide (5′-GGGCG**CACGGG**GCACT**CCCGTG**GTTCC-3’/3′-CCCGC**GTGCCC**CGTGA**GGGCAC**CAAGG-5′, boldface indicates the sequence of the E-boxes) was label with ^32^P. For binding reactions, the liver nuclear extracts were incubated first with 1.75 pmol of unlabeled mutated (one base deletion between E-boxes) LPK ChRE as a non-specific competitor (5′-GGGCG**CACGGG**GCCT**CCCGTG**GTTCC-3’/3′-CCCGC**GTGCCC**CGGA**GGGCAC**CAAGG-5′) for 10 min at room temperature. The wild-type LPK ChRE oligonucleotide at 0.035 pmol of ^32^P-labeled was added to the reaction mixture, followed by incubation for an additional 20 min. The use of the non-specific competitor eliminates non-specific DNA-binding signals [22]. The reaction mixture contained 10 mM Tris, pH 7.5, 50 mM NaCl, 1 mM MgCl_2_, 0.5 mM EDTA, 4% Glycerol, 0.5 mM DTT, and 50 μg/ml poly (dI-dC)•poly (dI-dC). The final mixture was then subjected with electrophoresis in a non-denaturing 4.5% polyacrylamide gel. The radioactive bands on the top portion of the gel, which represented the abundance of DNA shifted by ChREBP, were analyzed by exposing the gel on a storage phosphor plate overnight, followed by the scanning of autoradiograms in a Typhoon imager.

### 2.10 Statistical analysis

Data are shown as mean ± standard deviation. Prism 6.0 (GraphPad, Inc.) was used to perform the unpaired two-tailed student *t* test for comparison between any two groups. *p < 0.05, **p < 0.01 and ***p < 0.001 are considered statistically significant. Power analysis was done to analyze the results and number of mice in each experiment. All results fit the criteria of power (1-β error probability) > 0.95 with the minimal error probability of 0.05 (α error probability) at the given sample size indicated in the text. One-way ANCOVA analysis [23] was performed on energy expenditure and lean mass to assess the significance of differences between various treatment groups.

## 3 Results

### 3.1 PDK inhibitor PS10 preferentially stimulates PDC activity in liver and kidney

To assess the role of PDKs in regulating glucose and lipid metabolism we selected both PDK inhibitor PS10-treated DIO mice and PDK2/PDK4 DKO mice as models. The DKO genotype was established by the presence of a larger single 662-bp (from PDK2) and 806-bp (PDK4) fragments upon amplification of the genomic DNA (Supplementary Figure 1). Wild-type mice produced single 462-bp and 608-bp fragments for PDK2 and PDK4, respectively, against the corresponding larger transgene fragments in DKO mice.

**Figure 1:**
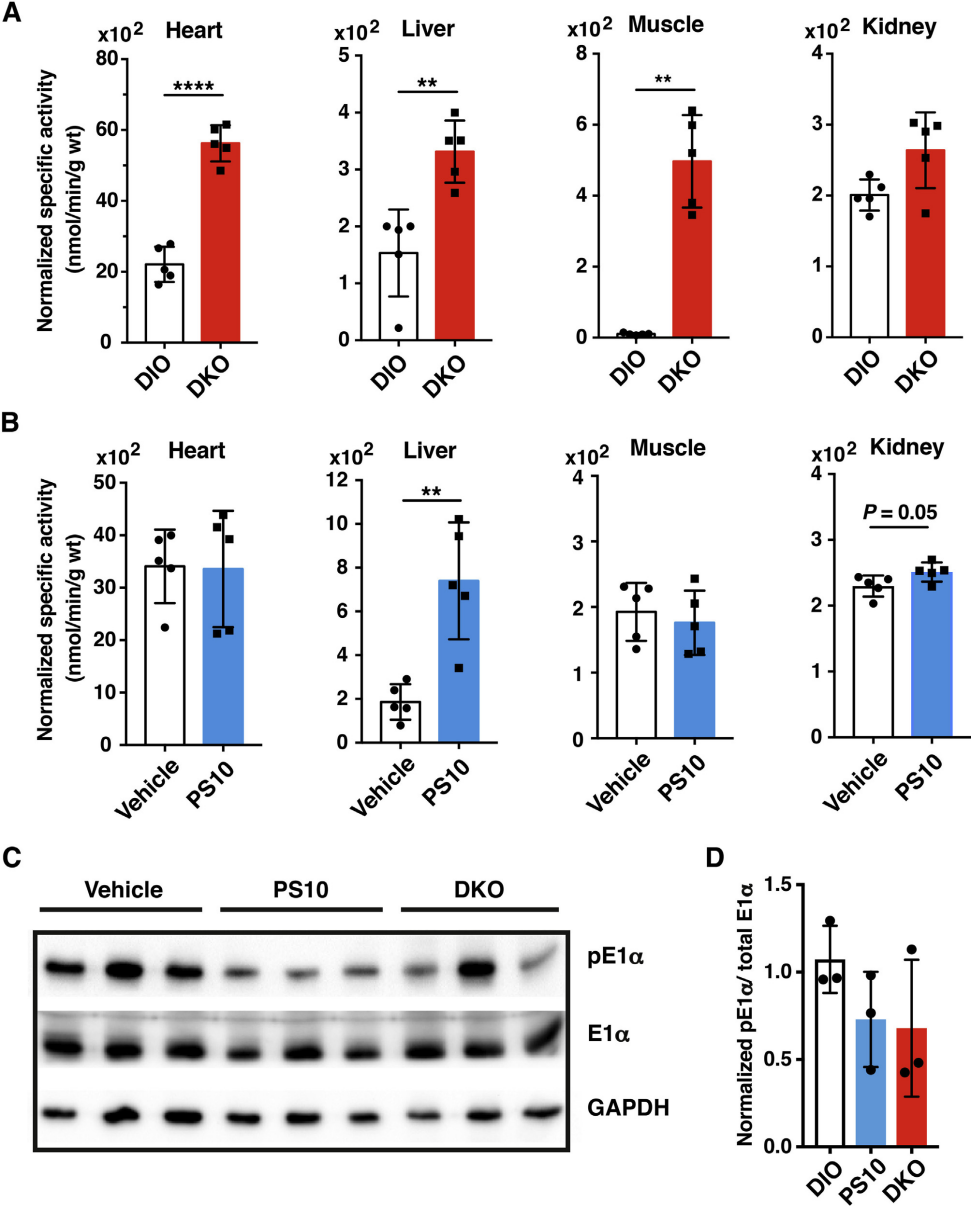
**Enhanced PDC activity in HFD-fed DKO mice and PS10-treated DIO mice**. Six-to eight-week old C57BL/6J male mice were randomized into two groups: vehicle- and PS10-treated. Prior to the treatment, mice were fed with a 60% high-fat diet, which contained 32% saturated and 68% unsaturated fat (catalog number: D12492, Research Diet Inc. New Brunswick, NJ) for 18 weeks to produce DIO mice. PS10 was dissolved in 100% DMSO and then diluted to make a 10% DMSO aqueous solution containing 17.5% (w/v) (2-hydroxypropyl)-β-cyclodextrin for delivery. Animals were dosed at 70 mg/kg intraperitoneally. At the end of the four-week treatment, animals were euthanized using carbon dioxide asphyxiation followed by cervical dislocation and dissection. A. PDC activity in different tissues of wild-type and DKO mice on HFD for 18 weeks (n = 6 in each group). B. Liver specific increase of PDC activity in DIO mice intraperitoneally treated with PS10 for four weeks (n = 5 in each group). Vehicle-treated DIO mice serve as a control. C. Western blots of liver samples showing reduced phosphorylation in the E1α subunit (pE1α) of PDC. The E1α antibody was obtained from MitoSciences/Abcam (Cambridge, MA). Antibodies against the phosphorylated serine (pSer293) residue of the E1α subunit were purchased from EMD Millipore/Calbiochem Biochemical (Billerica, MA). D. The ratio of the quantified pE1α: total E1α. Data are presented as mean ± S.D. *, *P* < 0.05, **, *P* < 0.01, ****, *P* < 0.0001.

Both 8-week old wild-type and DKO mice were fed a 60% high-fat diet (HFD) for 18 weeks. To simplify the nomenclature, the wild-type and DKO mice on the HFD are referred to as DIO and DKO mice, respectively, throughout the text. During the experimental period, DKO mice showed significantly lower weight gain than wild-type DIO mice (Supplementary Figure 2A). After 18 weeks on the HFD, wild-type DIO mice showed prominent hepatomegaly, which is essentially absent in DKO mice on the same HFD (Supplementary Figure 2B). These DIO mice were then treated with the PDK inhibitor PS10 at 70 mg/kg/day by intraperitoneal injection for four weeks, while DKO mice were maintained on the HFD without treatment. PS10 treatment led to a slight weight loss after three weeks of treatment (Supplementary Figure 2C). We treated DKO mice fed normal chow diet with PS10 at 70 mg/kg/day by IP injection for one week. Glucose tolerance tests were performed with both PS10-treated DKO mice and vehicle-treated DKO mice. We found no difference in glucose tolerance between these two groups. Thus, the results indicate that the drug is without effect in the DKO mice and that indeed PDK is the target (Supplementary Figure 3A). At the end of the four week HFD feeding, DKO mice show a systemic increase in PDC activity (heart, liver, muscle, and kidney) (Figure 1A). In contrast, preferential stimulation of PDC activity occurs in liver and to some extent kidney of PS10-treated relative to vehicle-treated DIO mice (Figure 1B). The increases in PDC activity results from reduced phosphorylation of the E1α subunit in the liver extracts of PS10-treated and DKO mice compared to the wild-type DIO control (Figure 1C). The ratio of phospho-E1α to total E1α is shown in Figure 1D. In a parallel study, wild-type DIO mice were treated with the classic PDK inhibitor DCA at 250 mg/kg/day for two weeks. At the end of the DCA treatment, PDC activity is significantly elevated in liver, heart, and muscle, supporting a systemic activation of PDC (Supplementary Figure 4A). Increased PDC activity is also associated with decreased phosphorylation of the E1α subunit (Supplementary Figure 4G).

### 3.2 Improved glucose, insulin, and pyruvate tolerance with reduced plasma insulin levels

**Figure 2:**
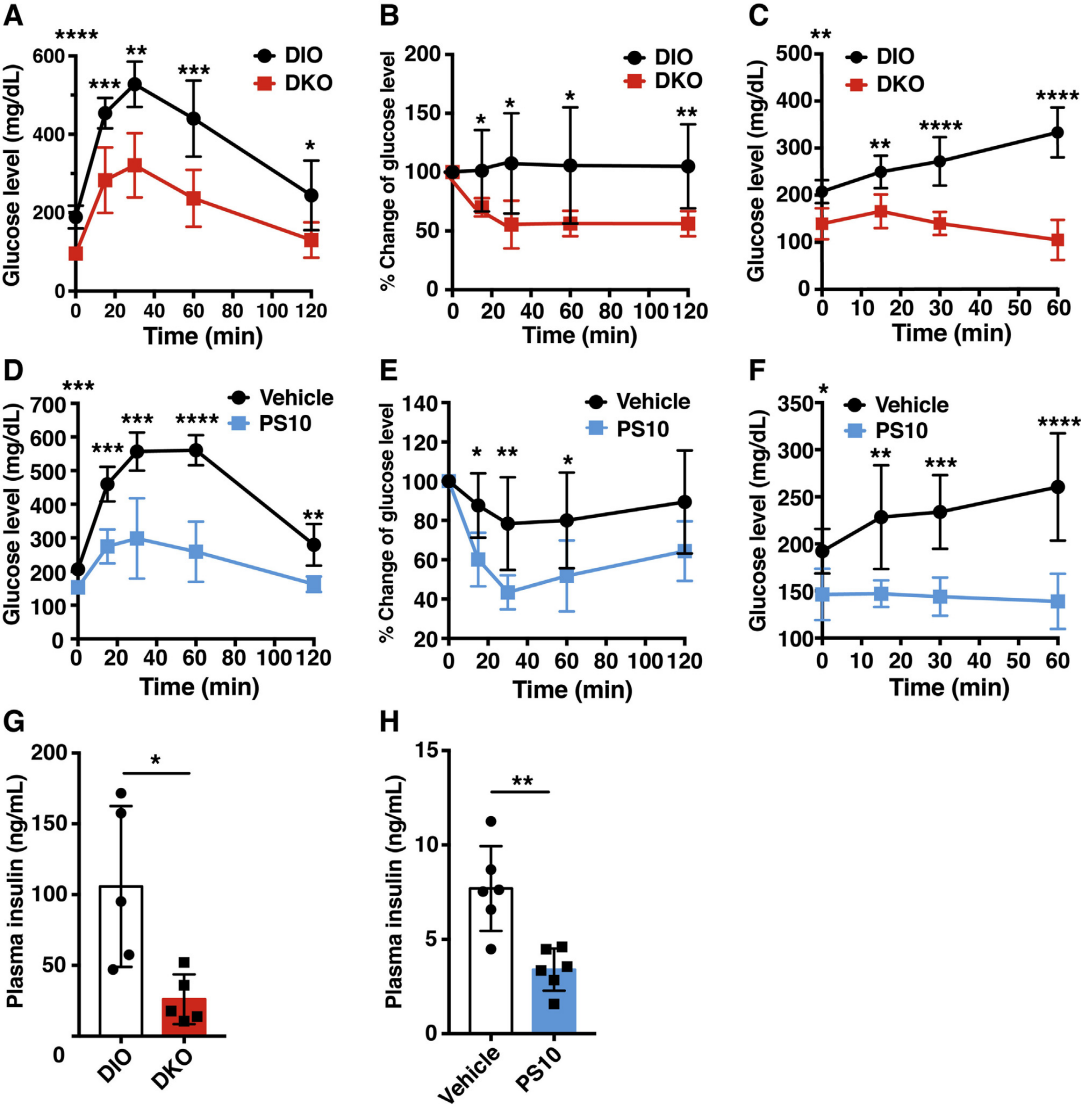
**Improved glucose tolerance, restored insulin sensitivity and reduced gluconeogenesis with HFD-fed DKO and PS10-treated DIO mice**. A. The glucose tolerance test with DKO and wild-type mice fed HFD, with the latter as controls. B. The insulin tolerance test with DKO mice. C. The pyruvate tolerance test with DKO mice (n = 5 in each group). D-F. The glucose, insulin, and pyruvate tolerance tests, respectively with vehicle- and PS10-treated DIO mice (n = 5 in each group). G. Reduced plasma insulin level in DKO mice (n = 5 in each group). H. Reduced plasma insulin level in PS10-treated DIO mice (n = 6 in each group). Data are presented as mean ± S.D. *, *P* < 0.05, **, *P* < 0.01, ***, P < 0.001, ****, *P* < 0.0001.

Compared to the DIO control, DKO mice on the HFD shows significantly improved glucose, insulin, and pyruvate tolerance tests (Figure 2A–C). Results of both glucose (Figure 2A) and insulin (Figure 2B) tolerance tests suggest increased insulin sensitivity in DKO mice. The decreased glucose levels upon pyruvate injection indicate reduced gluconeogenesis in DKO mice over the DIO control (Figure 2C). Similar results of improved glucose, insulin, and pyruvate tolerance tests were observed in PS10-treated compared to vehicle treated DIO mice (Figure 2D–F). The apparent increased insulin sensitivity is accompanied by lower plasma insulin concentrations in DKO mice than in wild-type DIO control (Figure 2G). Similarly, the plasma insulin level in PS10-treated DIO mice is lower, compared to that in vehicle-treated DIO mice (Figure 2H). The systemic augmentation of PDC activity also improves glucose tolerance (Supplementary Figure 4B) with a concomitant decrease in plasma insulin concentration (Supplementary Figure 4C) in DCA-treated DIO mice relative to the vehicle-treated DIO control.

### 3.3 Altered energy substrate utilization in DKO and PS10-treated DIO mice

**Figure 3:**
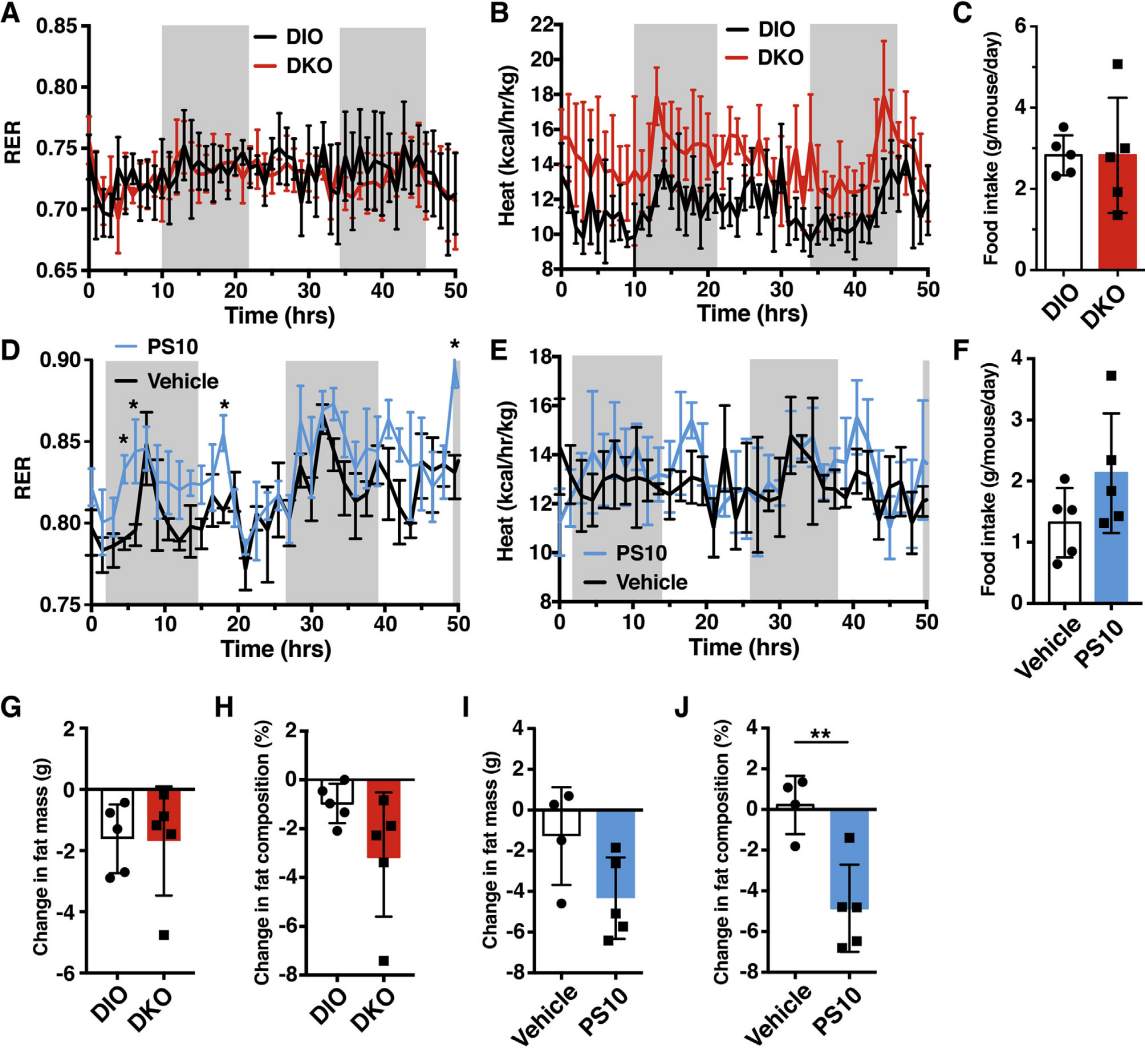
**Higher energy expenditure in DKO and** PS10-treated DIO mice. A. Similar respiratory exchange ratio (RER)s between HFD-fed DKO and wild-type mice. B. The energy expenditure of DKO mice verse wild-type mice on HFD. C. Unchanged food intake of DKO versus wild-type control mice (n = 5 in each group). D. Higher RER in PS10-treated mice verse vehicle-treated DIO mice. E. Higher energy expenditure in PS10-treated mice verse DIO mice. F. The lower food intake of PS10-treated mice relative to vehicle-treated DIO control mice (n = 5 in each group). G. Decreased fat mass composition in DKO mice versus wild-type DIO control mice (n = 6 in each group). H. Decreased fat mass composition in PS10-treated versus vehicle-treated DIO control mice (n = 5 in each group). Data are presented as mean ± S.D. *, *P* < 0.05, **, *P* < 0.01.

Metabolic cage studies for 5 days showed similar respiratory exchange rate (RER) in HFD-fed wild-type DIO and DKO mice (Figure 3A). However, heat production is higher in DKO mice than wild-type DIO mice (Figure 3B). The results are consistent with overall increases in both glucose and fatty acid oxidation. There was no difference in food intake between these DIO and DKO mice (Figure 3C). On the other hand, PS10-treated DIO mice showed higher RER than the vehicle-treated DIO control, indicating increased overall glucose over fatty acid oxidation in PS10-treated DIO mice (Figure 3D). The apparent increased glucose oxidation results in increased heat production in PS10-treated DIO mice over vehicle-treated (Figure 3E). One-way ANCOVA analysis [23] indicates that energy expenditure is significantly different between DIO versus DKO mice (F (1, 7) = 5.68, *P* = 0.048) and between vehicle-treated and PS10-treated mice (F (1,5) = 7.18, *P* = 0.043). Post hoc tests also show significant difference between DIO and DKO mice (*P* = 0.014) as well as between vehicle-treated and PS10-treated mice (*P* = 0.036). There is a trend for slightly higher food intake in PS10-treated DIO mice than in vehicle-treated (Figure 3F). Both DKO mice and the PS10-treated DIO mice showed decreased fat mass composition compared to their respective controls (wild-type DIO mice and vehicle-treated DIO mice, respectively) (Figure 3G–J). However, more significant fat mass and fat mass composition decrease is observed in PS10-treated DIO mice (Figure 3J).

### 3.4 Decreased glucose oxidation versus increased fatty acid oxidation in liver

**Figure 4:**
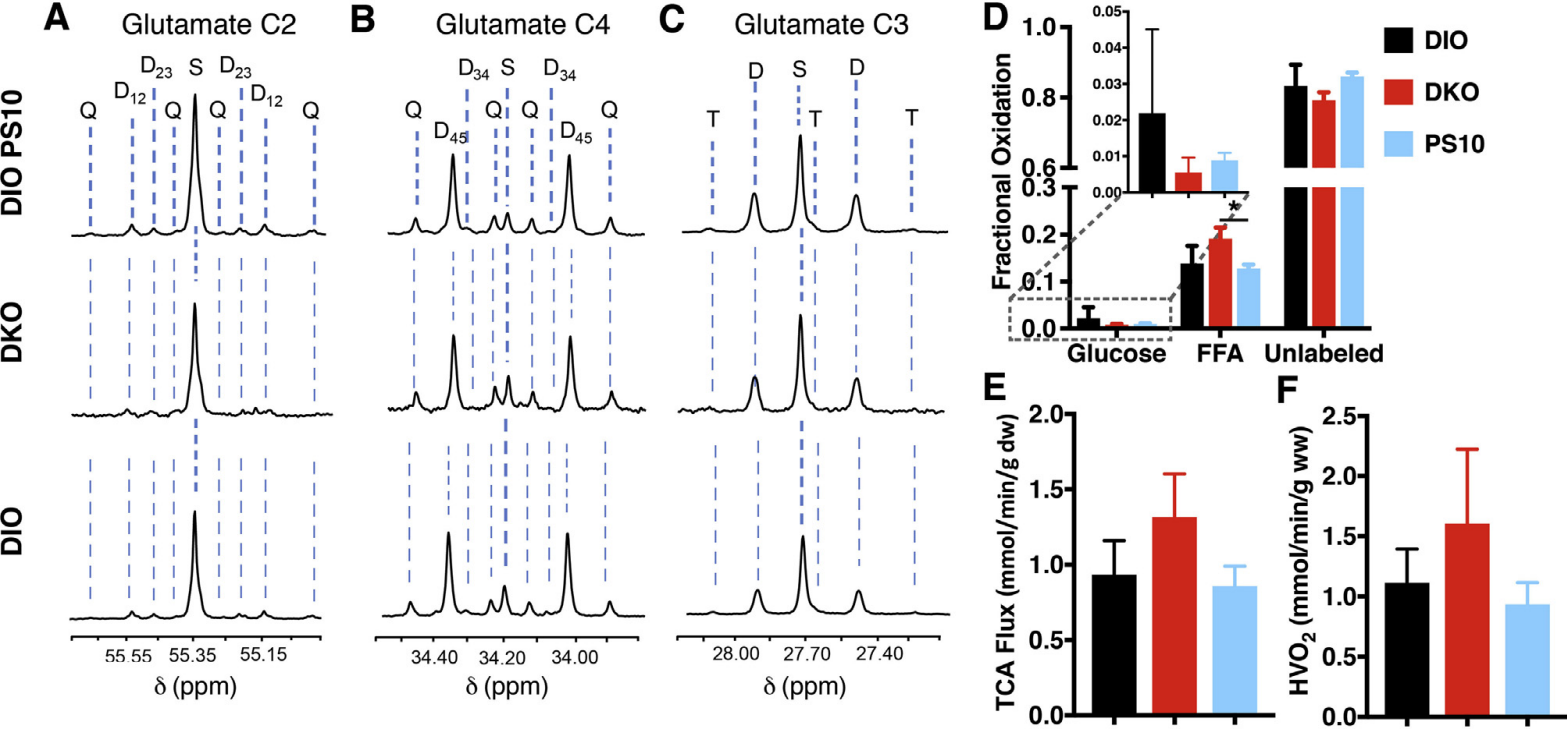
^**13**^**C NMR Isotopomer analysis from tissue extracts perfused isolated mice livers and flux analysis**. The following three groups (n = 3 per group) of DIO mice (6–8 week old) were studied: 1. DIO control, 2. DIO DKO, 3. PS10-treated DIO. Mice in DIO PS10-treated group was injected into the subcutaneous cavity with PS10 dose at 40–45 mg/kg. Mice were anesthetized by intramuscular injection of a ketamine/xylazine solution (0.1 mL, 85:15 w/w). Livers were cannulated via hepatic portal vein, isolated, connected to a perfusion column apparatus, and perfused at 25 cm H_2_O pressure with a modified Krebs-Henseleit (KH) buffer containing 5.5 mM [1,6–^13^C]glucose, 1.2 mM [3–^13^C]lactate, 0.12 mM [3–^13^C]pyruvate, 0.4 mM [U-^13^C] free fatty acids (FFA), and 0.75% BSA. The perfusion buffer for the PS10-treated group also contained 25 μL/L PS10. Oxygen consumption was measured at 25 min using blood gas analyzer (Instrumentation Laboratory, Lexington, MA) from the perfusate collected into a gas-tight syringe. Livers were perfused for 30 min before being freeze-clamped. The frozen tissues were pulverized in liquid nitrogen and extracted with perchloric acid (4–6%), neutralized, and reconstituted in D_2_O containing 1 mM EDTA and 0.5 mM 2,2-dimethyl-2-silapentane-5-sulfonate (DSS) standard. Proton-decoupled ^13^C-NMR spectra of heart extracts were acquired at Bruker 600 MHz spectrometer equipped with 10-mm CPDUL cryoprobe. Glutamate resonances were measured to determine the relative oxidation of [1,6–^13^C_2_]glucose, [U-^13^C]FFA, and unlabeled endogenous substrates (e.g., triglycerides and glycogen) and submitted as input for an isotopomer analysis using the program TCACALC v.2.07. A. Glutamate C-2 (55.35 ppm) B. Glutamate C-4 (34.20 ppm) and C. C-3 (27.60 ppm) spectra, D. Fractional Oxidation, E. TCA flux, and F. O_2_ consumption. HVO_2_, Hepatic venous oxygen. The letters S, D, T, and Q refer to a singlet, doublet (with the relevant J-coupled spins), triplet (a degenerate doublet of doublets) and quartet respectively. Data are presented as the mean ± SD (n = 3 per group) with significance indicated by “*” (*P* < 0.05).

Oxygen consumption of the DIO control livers measured under the metabolic steady state was 1.11 ± 0.28 μmol/min/g wet weight. To determine the effect of PDK in DKO and PS10 treatment on substrate utilization, the isolated liver were perfused with [1,6–^13^C_2_]glucose and [U-^13^C] long-chain fatty acid. The ^13^C multiplets from glutamate C-2 (Figure 4A), C-4 (Figure 4B), and C-3 (Figure 4C) in all three groups show very minimal changes. ^13^C NMR isotopomer analysis of liver extracts from DKO and PS-10-treated DIO mice showed a decrease in ^13^C-labeled glucose oxidation and a corresponding increase in labeled free fatty acid (FFA) oxidation comparing to the control livers (Figure 4D). Notably, PS10-treated DIO mice showed higher fractional hepatic glucose oxidation than DKO mice. Both PS-10-treated DIO (0.86 ± 0.13 μmol/min/g wet weight) and DKO (1.32 ± 0.29 μmol/min/g wet weight) livers had higher TCA cycle flux than the control livers (0.93 ± 0.22 μmol/min/g wet weight) (Figure 4E). Higher oxygen consumption was observed in the DKO livers (1.61 ± 0.62 μmol/min/g wet weight), while PS-10 treatment resulted in a smaller decrease in the oxygen consumption value (0.94 ± 0.18 μmol/min/g wet weight) (Figure 4F). These data demonstrate that livers oxidized more fatty acid than glucose for energy production in all three groups of the DIO mice. The PDK2/PDK4 DKO increased TCA cycle flux and the liver utilized more fatty acids, over glucose oxidation, to compensate for the increased energy demand. PS10 suppresses fatty acid oxidation in liver, which is in contrast to its augmentation in the DKO group.

### 3.5 Hepatic steatosis is significantly alleviated in DKO and PDK inhibitor-treated DIO mice

**Figure 5:**
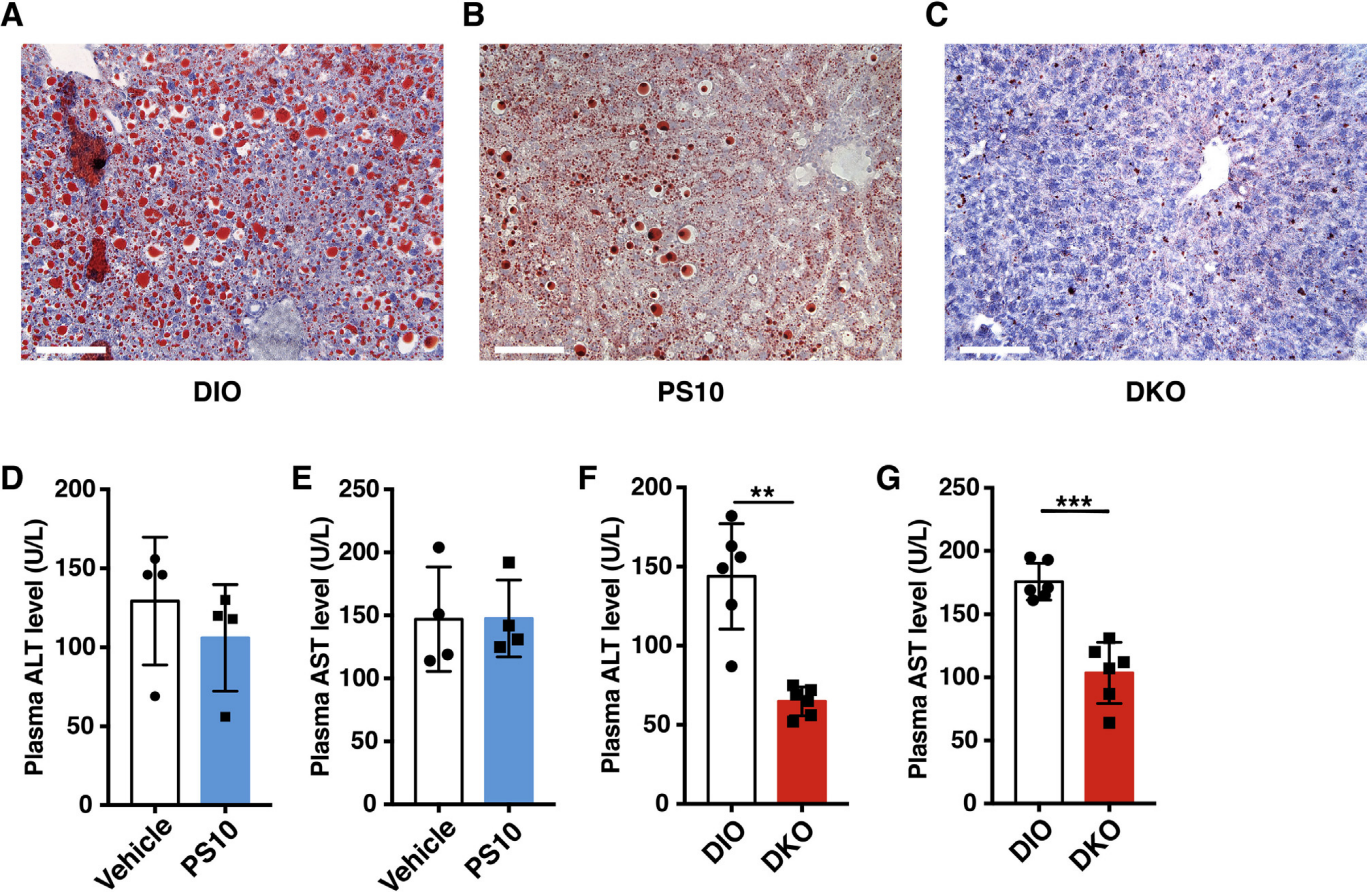
**Mitigation of hepatic steatosis in DKO and PS10-treated DIO mice**. A–C. Red Oil O staining of lipid on liver slides from the different study groups (scale bar is 100 μm). D. The plasma alanine aminotransferase (ALT) levels from PS10-treated DIO mice and DIO vehicle control mice. E. The plasma aspartate aminotransferase (AST) levels from PS10-treated DIO mice and DIO vehicle control mice (n = 5 in each group). F. The reduced plasma aspartate aminotransferase (ALT) levels from DKO mice versus wild-type DIO control mice. G. The reduced plasma alanine aminotransferase (AST) levels from DKO mice compared to wild-type DIO control mice (n = 6 in each group). Data are presented as mean ± S.D. **, *P* < 0.01, ***, *P* < 0.001.

Hepatic fat contents in vehicle-treated and PS10-treated DIO mice as well as DKO mice on the HFD were determined by Red Oil O stain. Macrovascular oil droplets were observed in livers from vehicle-treated DIO mice (Figure 5A). By comparison, reduced microvascular oil droplets are present in livers from PS10-treated animals (Figure 5B). Remarkably, only trace amounts of stained oil droplets can be seen in livers from DKO mice (Figure 5C). A systemic increase in PDC activity in various tissues (heart, liver, muscle and kidney) by IP dosing of DCA (250 mg/kg/day) for two weeks also led to reduced hepatic steatosis relative to vehicle-treated controls (Supplementary Figure 4H). However, the attenuation of hepatic lipid content is less prominent than those observed in PS10-treated DIO mice and DKO mice. The effects of reduced hepatic steatosis on liver function were also assessed. No significant differences in plasma alanine aminotransferase (ALT) (Figure 5D) and aspartate aminotransferase (AST) (Figure 5E) were detected between vehicle-treated and PS10-treated DIO mice. In contrast, plasma ALT (Figure 5F) and AST (Figure 5G) levels are significantly lower in DKO mice than in wild-type DIO control mice. The results indicate markedly improved liver functions, as a result of reduced hepatic steatosis in DKO mice, compared to wild-type DIO mice, and that PS10 does not have deleterious effects on the liver function.

### 3.6 Reduced plasma pyruvate/lactate and cholesterol and increased total ketone body concentrations in DKO mice during the fed state

**Figure 6:**
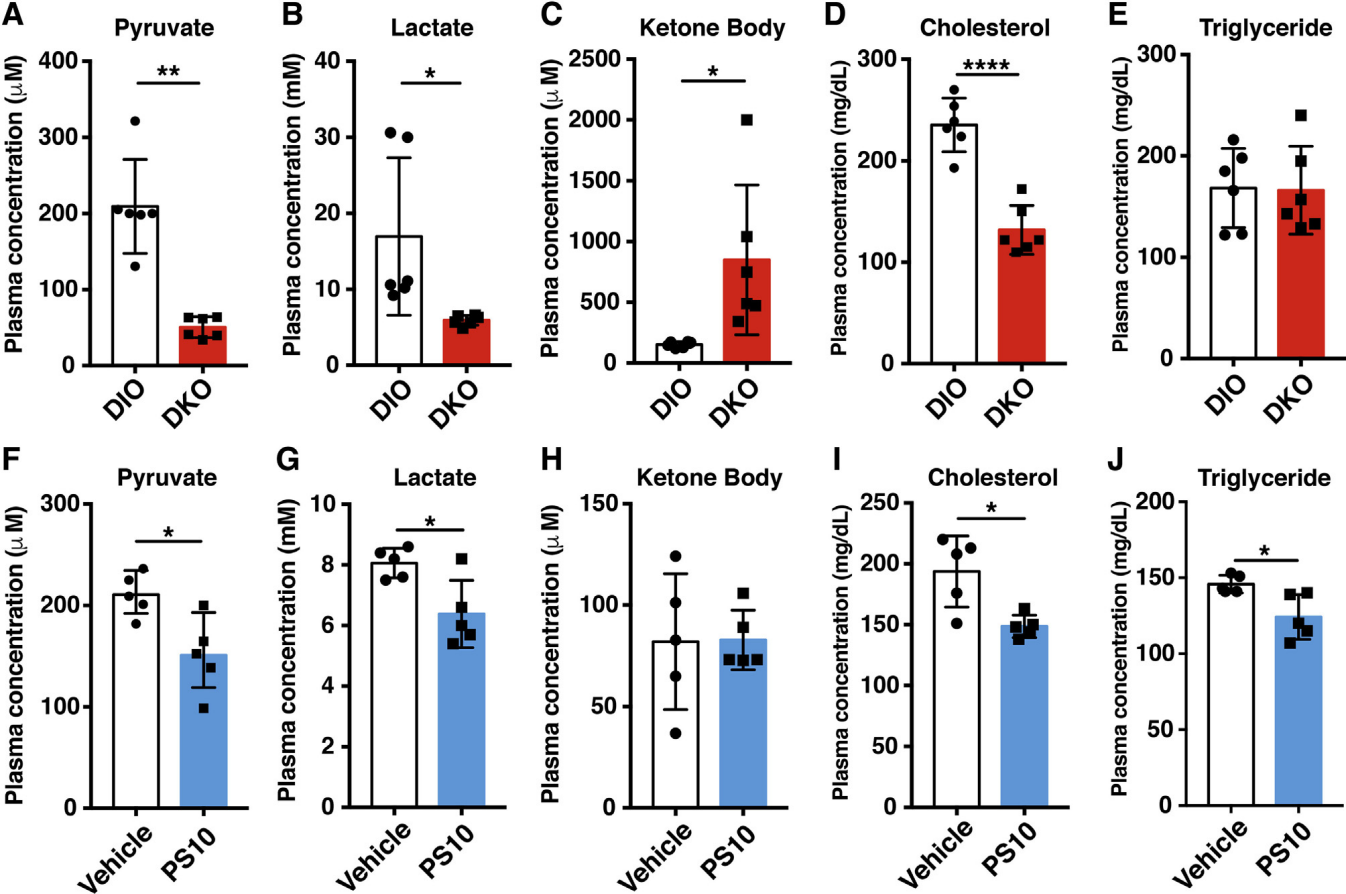
**The blood chemistry of HFD-fed DKO mice and PS10-treated DIO mice**. A. The pyruvate level in DKO mice. B. The lactate level in DKO mice. C. The total ketone body level in DKO mice. D. The cholesterol level in DKO mice. E. The triacylglyceride level in DKO mice (n = 6 in each group). F-J. The plasma metabolite levels shown in PS10-treated DIO mice (n = 5 in each group). All mice were in fed state when blood samples were obtained. Data are presented as mean ± S.D. *, *P* < 0.05, **, *P* < 0.01, ***, *P* < 0.001, ****, *P* < 0.0001.

The plasma pyruvate (Figure 6A) and lactate (Figure 6B) concentrations are significantly lowered in DKO mice compared to the wild-type DIO mice during the fed state. The DKO mice during the fed state show significantly higher total ketone body (Figure 6C) and lower plasma cholesterol concentrations (Figure 6D) than wild-type DIO mice. Similar triglyceride concentrations are present in DKO mice and the wild-type DIO control (Figure 5E). In parallel, lower pyruvate (Figure 6F) and lactate (Figure 6G) concentrations are present in PS10-treated DIO mice weighed against vehicle-treated. Notably, total ketone bodies are similar between PS10-treated and vehicle-treated DIO mice (Figure 5H). Plasma cholesterol (Figure 6I) and triglyceride (Figure 6J) concentrations are lowered in PS10-treated DIO mice than in vehicle-treated as reported previously [20]. DCA treatment recapitulates the DKO circulation metabolite results in DIO mice (Supplementary Figure 4C–D) as a result of systemic activation of PDC.

### 3.7 Targeting PDKs promotes AMPK inhibition of ChREBP-mediated lipogenesis in liver

**Figure 7:**
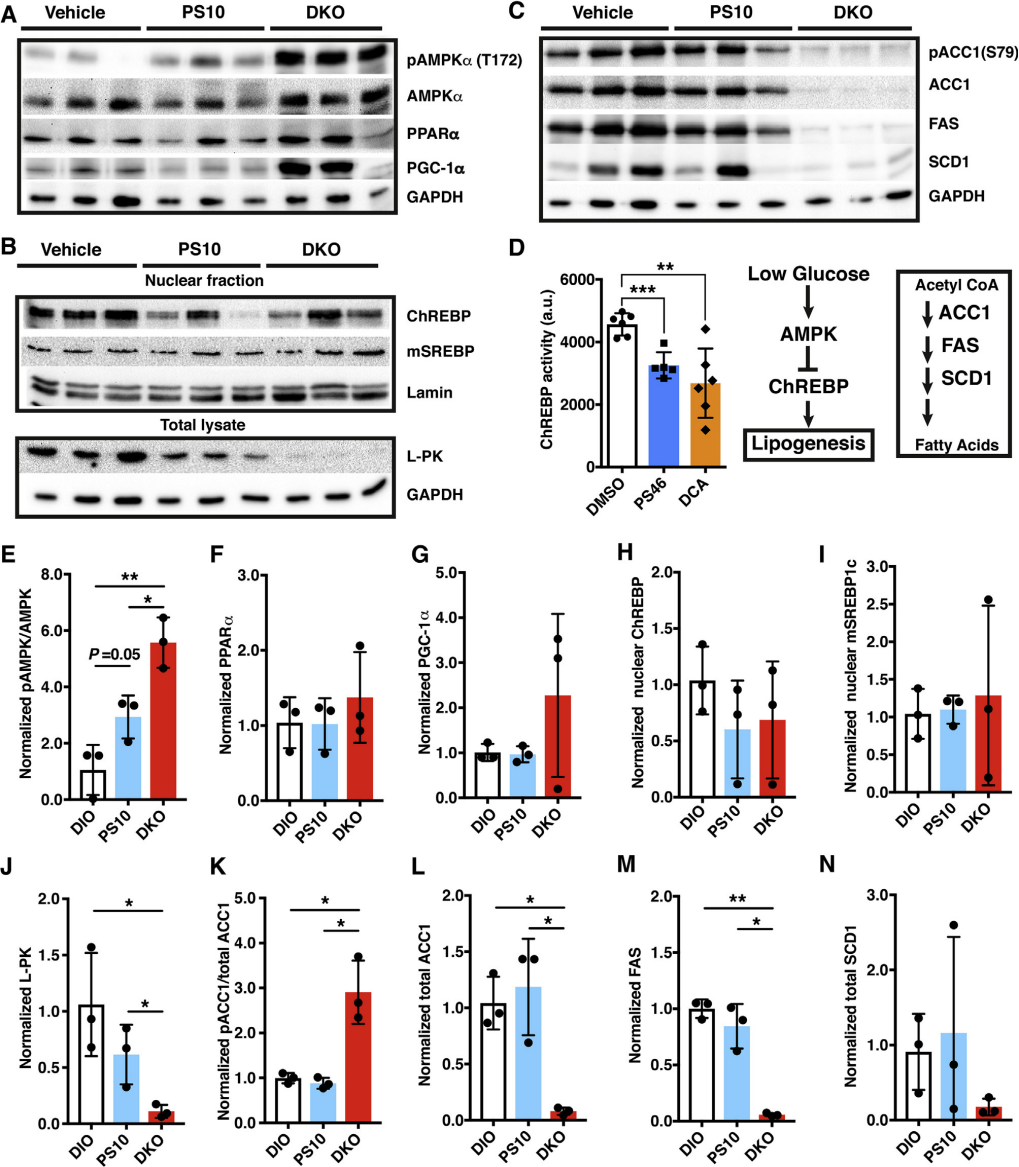
**Down-regulation of ChREBP-mediated hepatic lipogenesis in response to the lowering of glucose levels in DKO and PS10-treated DIO mice**. Western blots of key hepatic enzymes, panels A–C. A. Regulation of energy homeostasis, B. ChREBP signaling, C. lipogenesis. The sources of polyclonal antibodies were as follows: AMPK, pAMPK (Thr172), acetyl-CoA carboxylase 1 (ACC1), pACC1 (Ser79), fatty acid synthase (FAS), stearoyl-CoA desaturase 1 (SCD1), and lamin were purchased from Cell Signaling Technology (Danvers, MA); PGC1-α and SREBP1c (Thermo Fisher Scientific, Dallas, TX); GAPDH (Abcam, U.K.); ChREBP and PPARα (Santa Cruz, Waltham, MA); Liver-specific pyruvate kinase (L-PK) (Invitrogen, Carlsbad, CA). D. ChREBP activity in the nuclear extract, which reflects the binding to the carbohydrate-responsive element from L-PK. PS46 [(*S*)-3-amino-4-(4-((2-((2,4-dihydroxyphenyl)sulfonyl)isoindolin-5-yl)amino)piperidin-1-yl)-4-oxobutanamide] is an analogue of PS10, which inhibits all four PDK isoforms [21]. E to N. The quantification and ratios of band intensity from Western blots shown in panels A–C. Data are presented as mean ± S.D.*, *P* < 0.05, **, *P* < 0.01.

In PDK-deficient DKO mice on HFD, hepatic AMPK, pAMPK, PPAR-α and PGC1-α are uniformly elevated compared to vehicle-treated DIO mice on the same HFD (Figure 7A). Intermediate increases in pAMPK are also present in liver of PS10-treated DIO mice relative to the vehicle-treated DIO control (Figure 7E). The increased hepatic AMPK and pAMPK in PS10-treated and DKO mice are in parallel with lower plasma glucose levels (Figure 2A). The curtailed glucose levels in PS10-treated and DKO mice correlate with reduced ChREBP concentrations in hepatic nuclear fractions over vehicle-treated DIO mice (Figure 7B,H). The reduced nuclear ChREBP level in PS10-treated DIO mice and DKO mice are confirmed by diminished liver pyruvate kinase (L-PK) in the total lysates from livers (Figure 7B,J). The attenuated or absent cytosolic L-PK level serves as a specific marker for the reduced nuclear ChREBP activity under various physiological conditions [24]. In contrast, nuclear mature SREBP-1c levels remain unchanged in vehicle-treated control, PS10-treated DIO mice and DKO mice, all three groups being fed the same HFD (Figure 7B,I).

Nuclear ChREBP promotes insulin-independent lipogenesis in the liver [22]. The reduced nuclear ChREBP level correlates with essentially non-detectable protein concentrations of lipogenic enzymes ACC1, FAS and SCD1 in DKO mice on the HFD (Figure 7C). Moderately decreased lipogenic enzymes (ACC1, FAS and SCD1) are present in liver extracts from PS10-treated DIO mice, compared with the vehicle-treated control (Figure 7C).

### 3.8 Increased insulin signaling in PS10-treated DIO mice and HFD-fed DKO mice

**Figure 8:**
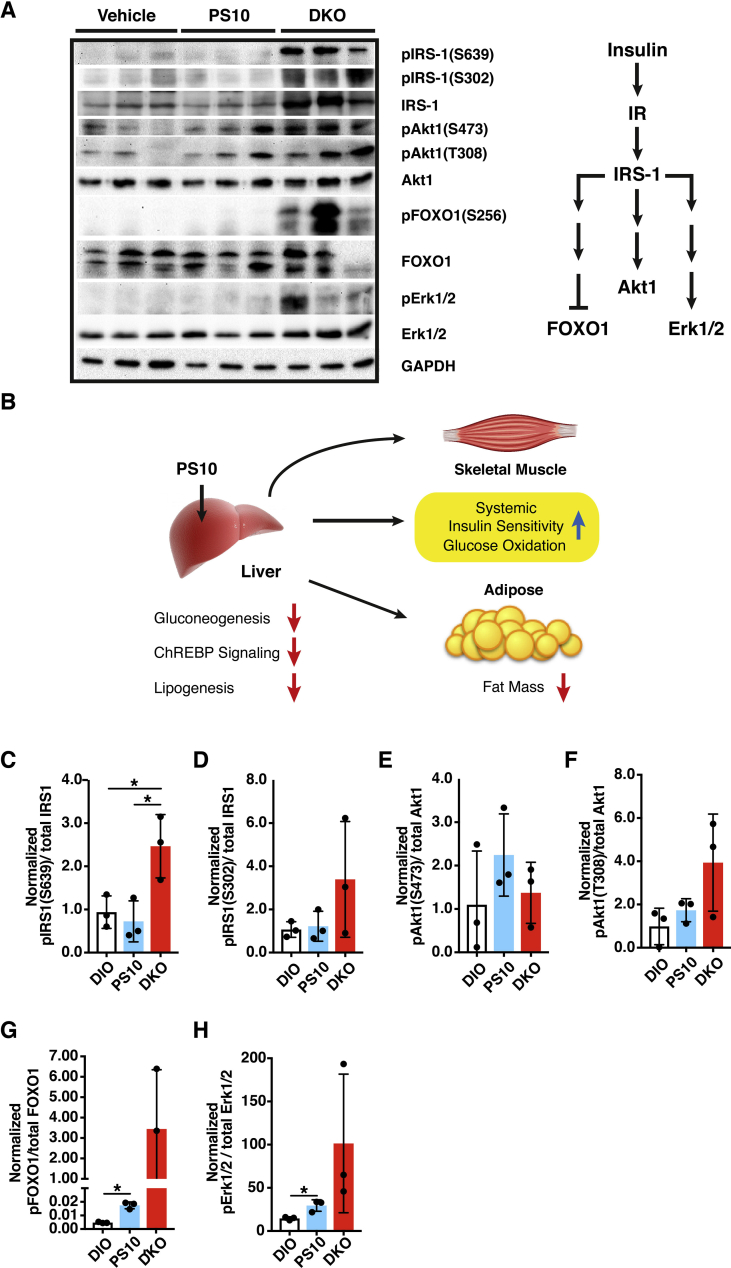
**Increased hepatic insulin signaling leads to restoration of systemic sensitivity in DKO and PS10-treated DIO mice**. A. Western blots of key modulators in the insulin signaling pathway, Polyclonal antibodies used were obtained from the following suppliers: insulin receptor substrate 1 (IRS1), pIRS-1 (Ser639), pIRS-1 (Ser302), Akt, pAkt (Ser473), pAkt (Thr 308), FOXO1, pFOXO1 (Ser256), Erk1/2, pErk1/2 (Thr202/Tyr204) (Cell Signaling Technology, Danvers, MA); GAPDH (Abcam, U.K.), B. A working model depicting liver-specific inhibition of PDKs in PS10-treated DIO mice. Decreased hepatic gluconeogenesis results in the lowering of plasma glucose concentrations, which down-regulates ChREBP signaling and lipogenesis in liver. Attenuated lipogenesis is manifested by lessened hepatic steatosis and reduced fat mass. Reduced glucose level leads to a restoration of systemic insulin sensitivity, notably in skeletal muscle. C to H. The quantification and ratios of western blot band intensity shown in panel A. Data are presented as mean ± S.D.*, *P* < 0.05.

In HFD-fed DKO mice, insulin receptor substrate-1 (IRS-1) and its phosphorylated counterparts pIRS-1 (S302) and pIRS-1 (S639) are markedly elevated, indicating a significantly higher insulin sensitivity over the vehicle-treated control DIO mice (Figure 8A,C and D). The elevated IRS-1 signaling in turn leads to increased phosphorylated forms of AKT1, i.e. pAKT1 (T308) and pAKT1 (S473) (Figure 8E,F). In a parallel pathway, increased IRS-1 expression (both non-phosphorylated and phosphorylated forms) results in phosphorylation and inactivation of FOXO-1 as represented by pFOXO-1 (S256) (Figure 8A,G). In another lateral pathway, increased IRS-1 expression and its phosphorylation are accompanied by increased phosphorylation and activation of Erk1 (T202) and Erk2 (Y204), as represented by pErk1/2 (Figure 8A,H).

## 4 Discussion

In our previous study [20], we observed marked differences in increased PDC activity between the acute and prolonged PS10 treatment. In the acute treatment, i.e. single injection, PDC activity in heart, liver, and kidney was invariably and robustly augmented. However, after a minimal 3-day treatment, the increase in PDC activity in heart and kidney became much less notable. We reason that heart and kidney do not utilize glucose as a major fuel source; therefore, these tissues might compensate for the effect from PS10 by partially restoring PDC activity. Liver is distinct in that it is the major gluconeogenic organ and is responsible for glucose cycling [25]. Therefore, the compensation of PDC activity does not occur in this organ. In addition, there are many efflux pumps to facilitate the active uptake of small molecules in the liver, which suggest effects of the PS10 treatment may be sustained better in the liver than other tissues. The detailed mechanisms for PS10 tissue specificity will require further investigation.

A key question regarding therapeutic approach to obesity and T2D is whether glucose or fatty acids are the primary fuel source. According to the glucose-fatty acid cycle, both fuel substrates compete for entry into the Krebs cycle for oxidative metabolism, where increased fatty acid oxidation likely suppresses glucose uptake and oxidation [26]. Our ^13^C NMR results showed that the fractional fatty acid oxidation outweighs the fractional glucose oxidation in livers from all three groups of the DIO control, DKO and PS10-treated DIO mice (Figure 4D). These findings are not unexpected, since all mouse groups used in the present study are on the same HFD. When fatty acids serve as the primary and preferred fuel source, fatty acid oxidation predominates in the liver [27], [28], [29]. The increased fatty acid oxidation is similar to that observed in the PDK2 knockout mice [30]. It has been suggested that a smaller pyruvate pool due to increased PDC activity leads to reduced anaplerosis in the TCA cycle, which triggers compensatory increased fatty acid oxidation [30]. Moreover, the increased TCA cycle flux (Figure 4E) in DKO mice causes the liver to utilize more fatty acids, over glucose oxidation, to meet the increased energy demand. On the other hand, the fraction of glucose oxidation is higher in PS10-treated DIO mice than in DKO mice; as a result, the portion of fatty acid oxidation is lower in the PS10-treated DIO mice compared to the DKO mice.

Go et al. [30] interpreted the lowering of TCA cycle intermediates to indicate a lower TCA cycle flux. However, the lower concentrations of TCA intermediates at the steady state do not necessarily correlate with lower metabolic fluxes. In contrast, we argue that intermediates dissipate more rapidly as a result of a higher TCA cycle flux. Given the tight correlation of the TCA cycle flux with oxygen consumption, our data suggest that the TCA cycle flux is increased in the DKO mice. Increased TCA cycle flux may boost energy production from acetyl-CoA, leading to the production of an acetyl-CoA sink, which along with enhanced fatty acid oxidation promote ketogenesis in the DKO liver. This notion is corroborated by our ^13^C tracer NMR studies (Figure 4) that show increased fatty acid oxidation in DKO mouse livers but not in PS10-treated livers. These NMR results are in agreement with markedly increased ketone body levels in the DKO mice (Figure 6C). As for the reason why increased glucose oxidation was not observed in DKO liver, further investigation is required.

With respect to the metabolic cage studies, no differences in RER values were obtained between DKO mice and the DIO control mice (Figure 3A). We interpret the results to indicate that fatty acid oxidation is increased in DKO mice to counter the effects from increased glucose oxidation, caused by a systemic increase in PDC activity, predominantly in skeletal muscle (Figure 1A). The increased heat expenditure in DKO mice compared to the wild-type DIO mice may also result from the systemic higher fractional oxidation of fatty acids over glucose (Figure 4D). On the other hand, the higher RER values in PS10-treated DIO mice than vehicle-treated DIO mice may reflect enhanced glucose oxidation, resulting from a robust increase in hepatic PDC activity (Figure 3D). These findings on fuel selection are consistent with liver being the principal glucose-producing organ and unable to utilize glucose efficiently as an energy source [24]. It follows that the lowering of plasma glucose concentrations in DKO and in PS10-treated DIO mice is not caused by increased hepatic glucose oxidation. Rather, the smaller pyruvate pools (Figure 6A,F) caused by increased hepatic PDC flux likely attenuates hepatic gluconeogenesis, resulting in lower glucose levels as indicated by improved pyruvate tolerance in DKO (Figure 2C) and PS10-treated DIO mice (Figure 2F).

In addition, because of lower circulating levels of insulin in DKO (Figure 2G) and PS10-treated DIO mice (Figure 2H), insulin sensitivity is restored in both animal groups as a result of tempered hyperinsulinemia (Figure 8). Increased insulin sensitivity results in restored metabolic flexibility and increased glucose oxidation upon insulin stimulation in peripheral tissues [31]. Enhanced insulin sensitivity further attenuates gluconeogenesis in liver [32]. Therefore, that pyruvate oxidation by PDC in peripheral tissues is likely increased, leading to enhanced pyruvate oxidation, rather than channeling pyruvate into glucose through increased gluconeogenesis in the liver.

It is well documented that increased PDC activity in PDK2 and PDK4 single knockout mice fed HFD leads to elevated ketone body production in the liver [11], [12]. The increased ketogenesis results from heightened fatty acid oxidation to maintain the TCA cycle flux [30]. Fasting further induces ketoacidosis and hypothermia in PDK2/PDK4 DKO mice due to inefficient ketone body oxidation [13]. In the present study, a significant increase in the plasma total ketone body is also present in the HFD-fed DKO mice (Figure 6C) but not PS10-treated DIO mice (Figure 6H). The increased fatty acid oxidation is supported by the elevated hepatic PPARα/PGC-1α levels in DKO mice (Figure 7A,F and G). On the other hand, the absence of increased PPARα/PGC-1α levels and heightened fatty acid oxidation in PS10-treated DIO mice explains the similar levels of total ketone body between the PS10-treated mice and the DIO control (Figure 6H).

The above results support the notion that liver-specific targeting of PDKs by treatment with the inhibitor PS10 in DIO mice is advantageous in that it poses no potentially deleterious ketoacidosis, as is the case with the HFD-fed DKO mice.

The transcription factor ChREBP promotes lipogenesis in response to increased hepatic glucose levels independent of insulin [22]. Glucose and cAMP down-regulate the L-PK gene through the phosphorylation of hepatic ChREBP by AMP-activated protein kinase (AMPK) [17] [33]. Here, the greater abundance of phosphorylated AMPK (pAMPK) indicates that the liver is responding to lower glucose levels in PS10-treated DIO mice as well as HFD-fed DKO mice (Figure 7A,E). The increased levels of pAMPK lead to the phosphorylation of ChREBP, preventing it from localizing to the nucleus, as indicated by the lower levels of ChREBP protein (Figure 7B,H) and the reduced activity (Figure 7D) in the hepatic nuclear fraction [33]. The reduction in cytosolic L-PK levels also responding to low glucose concentrations serves as a marker for decreased nuclear ChREBP protein and activity [33]. The dramatically lower L-PK expression (Figure 7B,J) also contributes to smaller pyruvate pools in DKO and PS10-treated DIO mice (Figure 4D). Significantly, the reduced nuclear ChREBP levels promote the down-regulation of lipogenic enzymes including ACC1, FAS, and SCD1. The phosphorylation on ACC1 (pACC1) at Ser79 by AMPK inhibits the activity of ACC1 [34]. Interestingly, the phospho-ACC1 over total ACC1 is strongly enhanced in DKO (Figure 7 K). These results indicate that the genetic knockout of PDKs not only inhibits the activity of ACC1 via AMPK, but also the expression of ACC1. In contrast, the nuclear insulin-dependent SREBP-1c remains unchanged in PS10-treated DIO and DKO mice compared to vehicle-treated DIO mice (Figure 7B,I). The above results, taken together, provide the first evidence that targeting hepatic PDKs with the PDK inhibitor, PS10, mitigates hepatic glucose production and attenuates ChREBP-mediated lipogenesis, resulting in markedly reduced hepatic steatosis. It should be noted that metformin also inhibits hepatic gluconeogenesis [35], [36]; however, it is still controversial as to whether metformin treatment alone causes persistent lowering of the liver fat content [10], [37]. It would appear that the PDK inhibitor PS10 possesses a significant advantage over metformin in preventing lipid accumulation and potential lipotoxicity. The mechanism by which low circulating glucose promotes AMPK phosphorylation by PDK deficiency or inhibition requires further investigation.

In conclusion, the present study demonstrates that targeting hepatic PDKs with the liver-specific inhibitor PS10 lowers plasma glucose concentration via reduced hepatic gluconeogenesis (Figure 8B). The lowering of glucose concentrations reduce plasma insulin levels and attenuate hepatic ChREBP signaling leading to reduced lipogenesis in liver in both PS10-treated and DKO mice. The net phenotype includes reduced hepatic steatosis, lower circulating cholesterol and triacylglyceride levels as well as reduced fat mass. The increased insulin signaling in the liver of PS10-treated DIO mice is communicated to other tissues, for example muscle, resulting in systemic restoration of insulin sensitivity and glucose oxidation. Therefore, our findings establish that specific targeting of hepatic PDKs with the PDK inhibitor PS10 is an effective therapeutic approach to obesity and T2D without the harmful ketoacidosis associated with systemic inhibition of PDKs.

## Appendix A Supplementary data

mmc1

## References

[1] Harris R.A., Bowker-Kinley M.M., Huang B., Wu P. (2002). Regulation of the activity of the pyruvate dehydrogenase complex. Advances in Enzyme Regulation.

[2] Zhang S., Hulver M.W., McMillan R.P., Cline M.A., Gilbert E.R. (2014). The pivotal role of pyruvate dehydrogenase kinases in metabolic flexibility. Nutrition and Metabolism (London).

[3] Rinnankoski-Tuikka R., Silvennoinen M., Torvinen S., Hulmi J.J., Lehti M., Kivela R. (2012). Effects of high-fat diet and physical activity on pyruvate dehydrogenase kinase-4 in mouse skeletal muscle. Nutrition and Metabolism (London).

[4] Rosa G., Di Rocco P., Manco M., Greco A.V., Castagneto M., Vidal H. (2003). Reduced PDK4 expression associates with increased insulin sensitivity in postobese patients. Obesity Research.

[5] Wu P., Inskeep K., Bowker-Kinley M.M., Popov K.M., Harris R.A. (1999). Mechanism responsible for inactivation of skeletal muscle pyruvate dehydrogenase complex in starvation and diabetes. Diabetes.

[6] Zhang M., Zhao Y., Li Z., Wang C. (2017). Pyruvate dehydrogenase kinase 4 mediates lipogenesis and contributes to the pathogenesis of nonalcoholic steatohepatitis. Biochemical and Biophysical Research Communications.

[7] Kwon H.S., Huang B., Unterman T.G., Harris R.A. (2004). Protein kinase B-alpha inhibits human pyruvate dehydrogenase kinase-4 gene induction by dexamethasone through inactivation of FOXO transcription factors. Diabetes.

[8] Furuyama T., Kitayama K., Yamashita H., Mori N. (2003). Forkhead transcription factor FOXO1 (FKHR)-dependent induction of PDK4 gene expression in skeletal muscle during energy deprivation. Biochemical Journal.

[9] Kim Y.D., Kim Y.H., Tadi S., Yu J.H., Yim Y.H., Jeoung N.H. (2012). Metformin inhibits growth hormone-mediated hepatic PDK4 gene expression through induction of orphan nuclear receptor small heterodimer partner. Diabetes.

[10] Lin H.Z., Yang S.Q., Chuckaree C., Kuhajda F., Ronnet G., Diehl A.M. (2000). Metformin reverses fatty liver disease in obese, leptin-deficient mice. Nature Medicine.

[11] Hwang B., Jeoung N.H., Harris R.A. (2009). Pyruvate dehydrogenase kinase isoenzyme 4 (PDHK4) deficiency attenuates the long-term negative effects of a high-saturated fat diet. Biochemical Journal.

[12] Jeoung N.H., Harris R.A. (2008). Pyruvate dehydrogenase kinase-4 deficiency lowers blood glucose and improves glucose tolerance in diet-induced obese mice. American Journal of Physiology, Endocrinology and Metabolism.

[13] Jeoung N.H., Rahimi Y., Wu P., Lee W.N., Harris R.A. (2012). Fasting induces ketoacidosis and hypothermia in PDHK2/PDHK4-double-knockout mice. Biochemical Journal.

[14] Horton J.D., Goldstein J.L., Brown M.S. (2002). SREBPs: transcriptional mediators of lipid homeostasis. Cold Spring Harbor Symposia on Quantitative Biology.

[15] Browning J.D., Horton J.D. (2004). Molecular mediators of hepatic steatosis and liver injury. Journal of Clinical Investigation.

[16] Uyeda K., Repa J.J. (2006). Carbohydrate response element binding protein, ChREBP, a transcription factor coupling hepatic glucose utilization and lipid synthesis. Cell Metabolism.

[17] Filhoulaud G., Guilmeau S., Dentin R., Girard J., Postic C. (2013). Novel insights into ChREBP regulation and function. Trends in Endocrinology and Metabolism.

[18] Herman M.A., Peroni O.D., Villoria J., Schon M.R., Abumrad N.A., Bluher M. (2012). A novel ChREBP isoform in adipose tissue regulates systemic glucose metabolism. Nature.

[19] Eissing L., Scherer T., Todter K., Knippschild U., Greve J.W., Buurman W.A. (2013). De novo lipogenesis in human fat and liver is linked to ChREBP-beta and metabolic health. Nature Communications.

[20] Tso S.C., Qi X., Gui W.J., Wu C.Y., Chuang J.L., Wernstedt-Asterholm I. (2014). Structure-guided development of specific pyruvate dehydrogenase kinase inhibitors targeting the ATP-binding pocket. Journal of Biological Chemistry.

[21] Tso S.C., Lou M., Wu C.Y., Gui W.J., Chuang J.L., Morlock L.K. (2017). Development of dihydroxyphenyl sulfonylisoindoline derivatives as liver-targeting pyruvate dehydrogenase kinase inhibitors. Journal of Medicinal Chemistry.

[22] Yamashita H., Takenoshita M., Sakurai M., Bruick R.K., Henzel W.J., Shillinglaw W. (2001). A glucose-responsive transcription factor that regulates carbohydrate metabolism in the liver. Proceedings of the National Academy of Sciences of the U S A.

[23] Tschop M.H., Speakman J.R., Arch J.R., Auwerx J., Bruning J.C., Chan L. (2011). A guide to analysis of mouse energy metabolism. Nature Methods.

[24] Burgess S.C., Iizuka K., Jeoung N.H., Harris R.A., Kashiwaya Y., Veech R.L. (2008). Carbohydrate-response element-binding protein deletion alters substrate utilization producing an energy-deficient liver. Journal of Biological Chemistry.

[25] Efendic S., Wajngot A., Vranic M. (1985). Increased activity of the glucose cycle in the liver: early characteristic of type 2 diabetes. Proceedings of the National Academy of Sciences of the U S A.

[26] Randle P.J., Garland P.B., Hales C.N., Newsholme E.A. (1963). The glucose fatty-acid cycle. Its role in insulin sensitivity and the metabolic disturbances of diabetes mellitus. Lancet.

[27] Neat C.E., Thomassen M.S., Osmundsen H. (1981). Effects of high-fat diets on hepatic fatty acid oxidation in the rat. Isolation of rat liver peroxisomes by vertical-rotor centrifugation by using a self-generated, iso-osmotic, Percoll gradient. Biochemical Journal.

[28] Cole M.A., Murray A.J., Cochlin L.E., Heather L.C., McAleese S., Knight N.S. (2011). A high fat diet increases mitochondrial fatty acid oxidation and uncoupling to decrease efficiency in rat heart. Basic Research in Cardiology.

[29] Schrauwen P., Wagenmakers A.J., van Marken Lichtenbelt W.D., Saris W.H., Westerterp K.R. (2000). Increase in fat oxidation on a high-fat diet is accompanied by an increase in triglyceride-derived fatty acid oxidation. Diabetes.

[30] Go Y., Jeong J.Y., Jeoung N.H., Jeon J.H., Park B.Y., Kang H.J. (2016). Inhibition of pyruvate dehydrogenase kinase 2 protects against hepatic steatosis through modulation of tricarboxylic acid cycle anaplerosis and ketogenesis. Diabetes.

[31] Kelley D.E., Goodpaster B., Wing R.R., Simoneau J.A. (1999). Skeletal muscle fatty acid metabolism in association with insulin resistance, obesity, and weight loss. American Journal of Physiology.

[32] Michael M.D., Kulkarni R.N., Postic C., Previs S.F., Shulman G.I., Magnuson M.A. (2000). Loss of insulin signaling in hepatocytes leads to severe insulin resistance and progressive hepatic dysfunction. Molecular Cell.

[33] Kawaguchi T., Osatomi K., Yamashita H., Kabashima T., Uyeda K. (2002). Mechanism for fatty acid “sparing” effect on glucose-induced transcription: regulation of carbohydrate-responsive element-binding protein by AMP-activated protein kinase. Journal of Biological Chemistry.

[34] Ha J., Daniel S., Broyles S.S., Kim K.H. (1994). Critical phosphorylation sites for acetyl-CoA carboxylase activity. Journal of Biological Chemistry.

[35] Yasuda N., Inoue T., Nagakura T., Yamazaki K., Kira K., Saeki T. (2004). Metformin causes reduction of food intake and body weight gain and improvement of glucose intolerance in combination with dipeptidyl peptidase IV inhibitor in Zucker fa/fa rats. Journal of Pharmacology and Experimental Therapeutics.

[36] Kim Y.D., Park K.G., Lee Y.S., Park Y.Y., Kim D.K., Nedumaran B. (2008). Metformin inhibits hepatic gluconeogenesis through AMP-activated protein kinase-dependent regulation of the orphan nuclear receptor SHP. Diabetes.

[37] Tang W., Xu Q., Hong T., Tong G., Feng W., Shen S. (2016). Comparative efficacy of anti-diabetic agents on nonalcoholic fatty liver disease in patients with type 2 diabetes mellitus: a systematic review and meta-analysis of randomized and non-randomized studies. Diabetes/Metabolism Research and Reviews.

