# Supplementary material for: Targeting hepatic pyruvate dehydrogenase kinases restores insulin signaling and mitigates ChREBP-mediated lipogenesis in diet-induced obese mice
Source: Mol Metab. 2018 Mar 31;12:12–24. doi: 10.1016/j.molmet.2018.03.014 (PMC6001905; doi:10.1016/j.molmet.2018.03.014)
Supplement: mmc1 [file mmc1.docx]

**Supplementary Figure 1. Genotyping results of DKO mice.** A. The PDK2 knockout genotype. The expected 662-bp (PDK2 knockout) and 462-bp (wild-type) DNA fragments are depicted. B. The PDK4 knockout genotype. The expected DNA fragments of 806-bp (PDK4 knockout) and 608-bp (wild-type) are depicted.

**
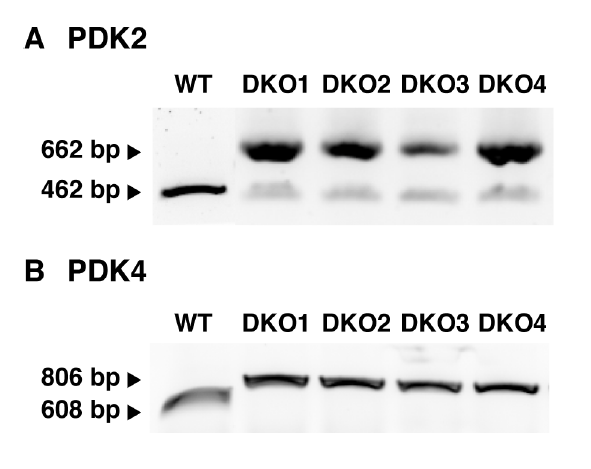
**

**Supplementary Figure 2. Reduced total body weight and absence of hepatomegaly in HFD-fed DKO mice compared to wild-type DIO control.** A. The growth curves show significantly slower total body weight gains in DKO mice than in wild-type DIO mice. (n = 11 in each group). The data are presented as mean ± SD. B. DKO mouse showing essentially absence of the prominent hepatomegaly manifested by wild-type (WT) control after HFD feeding for 18 weeks. C.  Weight curves for three-week PS10-treated versus vehicle-treated mice.

**Supplementary Figure 3. PS10 treatment is without effect on glucose tolerance in DKO mice.** The glucose tolerance test with DKO mice fed a normal chow diet. Mice were treated with PS10 at 70mg/kg/day for one week. No difference in glucose tolerance was observed between the PS10-treated group (n = 7) and vehicle-treated (n = 6), indicating that PDK are the target

.
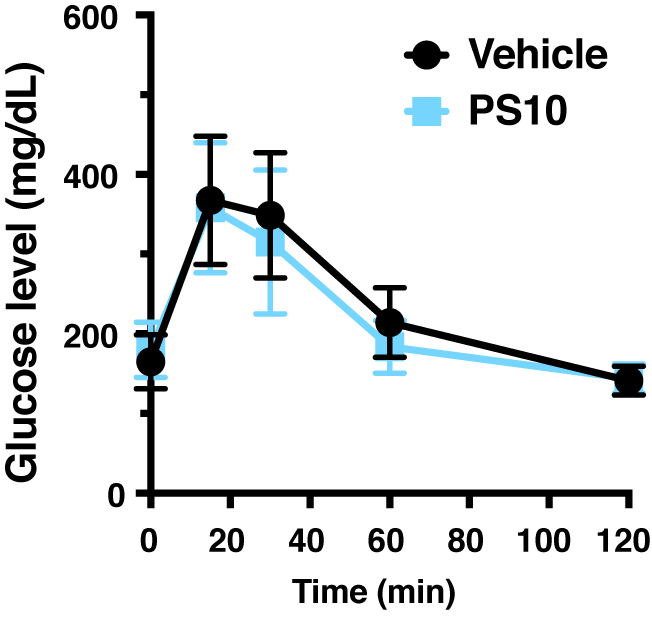


**Supplementary Figure 4. Systemic enhancement of PDC activity by DCA improves glucose tolerance but increases total ketone body concentrations.** A. Systemic elevation of PDC activity in tissues from DIO mice treated with DCA for two weeks at 250 mg/kg/day intraperitoneally. B. The glucose tolerance test with vehicle- and DCA-treated DIO mice. C-F. Reduced plasma insulin, lactate, total ketone body and cholesterol levels in DCA-treated DIO mice compared to vehicle-treated DIO controls (n = 6 in each group). G. Western blot of phosphorylation of PDC E1α subunit, ACC1, FAS, L-PK pAMPK, AMPK and GAPDH in vehicle-treated and DCA-treated DIO mice. H. The representative Oil Red O staining of liver slides from vehicle- and DCA-treated DIO mice (scale bar is 100μm). Data is presented as mean ± S.D. I – M. The quantification and ratios of band intensity from Western blots shown in panel G. *, *P* < 0.05, **, *P* <0.01, ***, *P* < 0.001. I-M. Normalized integration of pE1α/E1α, pAMPK/AMPK, ACC1, FAS, and L-PK, respectively.
